# Iquitos Virus in Traveler Returning to the United States from Ecuador

**DOI:** 10.3201/eid3011.240708

**Published:** 2024-11

**Authors:** Katherine Baer, Itika Arora, Jayden Kimbro, Ali Haider, Michelle Mott, Kyleigh Marshall, Henry M. Wu, Jessica Fairley, Anne Piantadosi, David R. Myers, Jesse J. Waggoner

**Affiliations:** Emory TravelWell Center, Atlanta, Georgia, USA (K. Baer); Emory University School of Medicine, Atlanta (K. Baer, I. Arora, J. Kimbro, A. Haider, M. Mott, H.M. Wu, J. Fairley, A. Piantadosi, J.J. Waggoner); Emory University Rollins School of Public Health, Atlanta (K. Marshall, J. Fairley, J.J. Waggoner); Emory University, Atlanta (D.R. Myers); Georgia Institute of Technology, Atlanta (D.R. Myers); American and Asian Centers for Arboviral Research and Enhanced Surveillance, Atlanta (J.J. Waggoner)

**Keywords:** Iquitos virus, Oropouche virus, viruses, returned traveler, Ecuador, RNAES, United States

## Abstract

We describe the case of a returned traveler to the United States from Ecuador who had an acute febrile illness, initially diagnosed as Oropouche fever. This illness was later confirmed to be a rare infection with Iquitos virus, a related bunyavirus that shares 2 of 3 genome segments with Oropouche virus.

Oropouche virus (OROV) is a species in the Simbu serogroup of bunyaviruses (genus *Orthobunyavirus*) that includes the reassortant species Iquitos virus (IQTV) and Madre de Dios virus. The species share common small and large genome segments but differ in the medium segment (*1*,*2*). Symptomatic human infections with the viruses typically manifest with nonspecific signs and symptoms (e.g., fever, headache, myalgias, and arthralgias) that cannot be clinically differentiated from other common tropical febrile illnesses such as dengue, malaria, and leptospirosis (*2*–*4*). 

In 2024, a large OROV outbreak has affected countries of the Amazon River Basin, with cases occurring in Brazil, Peru, Colombia, and Bolivia (*5*). However, despite previous detection of OROV transmission in Ecuador in 2016–2017 (*3*), cases have not been reported to the Pan American Health Organization from Ecuador in 2024 (*5*).

In early April 2024, a 38-year-old man with no notable medical history visited a healthcare system in Atlanta, Georgia, USA, with an acute febrile illness after returning from Ecuador. During a 10-day itinerary, he visited the capital city of Quito, Esmeraldas Province in the northwest, and Napo Province in the Amazon Basin (Figure 1), where he noted numerous bug bites. The patient did not take malaria prophylaxis but consistently used a DEET-containing insect repellant during the trip. He had 1 day of diarrhea on his last day in Ecuador; then, 2 days after returning to the United States, he had fevers reaching 102°F and chills, sweats, headache, and pain with eye movement. He was seen in an emergency department and was found to have a slight elevation in his creatinine (1.3 mg/dL [reference range 0.6-1.2 mg/dL]) but otherwise normal complete metabolic panel and complete blood count results. He was discharged home with an outpatient referral to the Emory TravelWell Center (also in Atlanta) the following day. 

**Figure 1:**
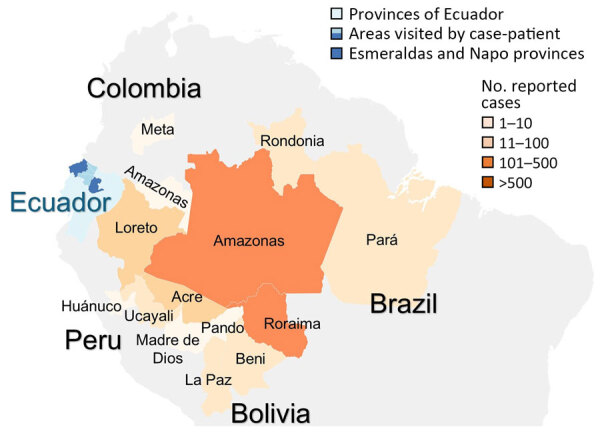
Provinces of Ecuador (light blue), including areas visited by a traveler from the United States who was infected with Iquitos virus (medium and dark blue); the provinces of Esmeraldas and Napo (dark blue) highlighted as areas of highest concern for contracting the virus. Surrounding provinces with confirmed Oropouche virus infection cases in Brazil, Colombia, Peru, and Bolivia are shown; shading indicates the number of reported cases, based on data from the Pan American Health Organization (*5*).

During that visit, he reported fatigue, sleeping 12–14 hours a day, and numerous itchy “bug bites” on his arms and ankles. Examination revealed he had unremarkable vital signs and a diffuse papular rash across his forearms and lower legs. The patient’s illness was reported in the GeoSentinel database (*6*), and he consented to the collection and testing of EDTA whole blood and serum as part of a research study into the causes of infection in returned travelers. At a follow-up visit the next week, he reported that the fever had resolved after 2 days and that the headache and fatigue resolved over 10 days. The rash initially evolved to hyperpigmented plaques and then resolved with topical hydrocortisone and diphenhydramine.

We processed whole blood and serum with a laboratory-developed nucleic acid extraction and storage protocol (i.e., the RNA extraction and storage [RNAES] protocol) (*7*). All eluates were negative for Zika, chikungunya, and dengue viruses on a laboratory-developed assay and negative for *Leptospira* and *Plasmodium* species (*8*). Eluates from serum and whole blood tested positive in a laboratory-developed real-time reverse transcription PCR (RT-PCR) that targets the small genome segment of OROV and related bunyaviruses (Appendix Figure, panel A) (*4*). We confirmed this finding by reextraction and retesting of an aliquot of whole blood using a second real-time RT-PCR targeting a different portion of the small genome segment (Appendix Figure, panel B) (*9*).

We successfully generated partial sequences for the coding regions of the small (83%), medium (27%), and large (37%) segments (GenBank accession nos. PQ325301–4) (Appendix). Phylogenetic analysis indicated that the small and large segments from the returned traveler were most closely related to an Oropouche virus sample obtained in Ecuador in 2016, and they clustered just basal to sequences from samples obtained from Brazil in 2023 (Figure 2, panel A, B). However, phylogenetic analysis of the medium segment confirmed that it was most closely related to IQTV, the only other available sequences of which were from Peru (Figure 2, panel C).

**Figure 2:**
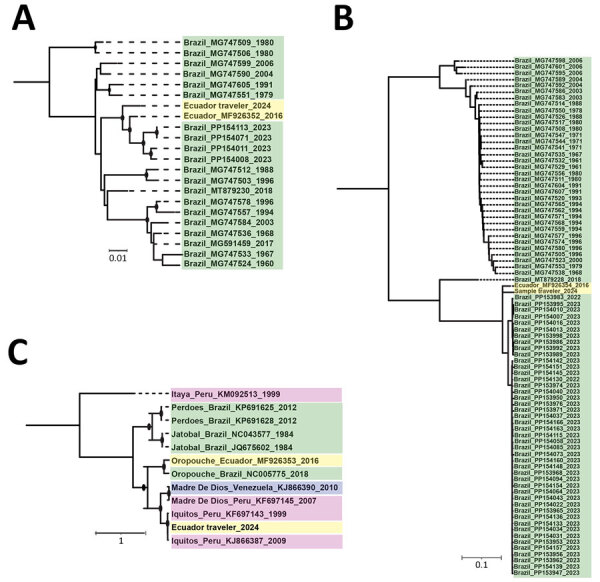
Maximum-likelihood phylogenetic analyses of the small segment (A), large segment (B), and medium segment (C) of Iquitos virus from a traveler returning to the United States from Ecuador. Sequences from the study have been deposited into GenBank (accession nos. PQ325301–4); reference sequences were obtained from National Center for Biotechnology Information Virus database. Panels A and B contain all available complete Oropouche small and large virus sequences, after removing identical sequences; panel C contains all available complete medium sequences for Iquitos, Oropouche, Itaya, Jatobal, Madre de Dios, and Perdoes viruses. Nodes with black circles have ultrafast bootstrap values >90. Sequence names are color-coded according to country of origin. Nucleotide substitution models were as follows: small segment, transversion model with empirical base frequencies and a gamma distribution of rates with 4 categories and α = 0.081; medium segment; transition model with empirical base frequencies and a gamma distribution of rates with 4 categories and α = 5.156; and large segment: general time-reversible model with empirical base frequencies, allowing for invariant sites and a gamma distribution of rates with 2 categories and α = 0.125. Scale bars indicate number of nucleotide substitutions per site.

Fever in a returned traveler can result from myriad etiologies that may be unfamiliar to providers in nonendemic areas and for which diagnostic testing is often limited (*6*). For the case we describe, systematic screening tools and economical laboratory solutions enabled the initial detection of OROV or a related bunyavirus, which has important implications for clinical management, given that meningitis and relapsing disease have been reported in OROV infection (*2*). However, further characterization by next-generation sequencing identified this virus as IQTV, a related bunyavirus that also circulates in the Amazon Basin and may have contributed to reassortment events that led to current OROV genetic diversity in South America (*10*). IQTV reportedly causes a clinical illness similar to Oropouche fever; of note, however, infection with OROV does not appear to protect against future IQTV infection (*1*). Finally, this case provides support for increased bunyavirus monitoring in Ecuador (*3*), where these viruses may have gone undetected or underreported because of limited diagnostics, poor healthcare access, sociopolitical instability, or a combination of those factors.

Appendix Additional information about Iquitos virus in traveler returning to the United States from Ecuador.
