# Supplementary material for: Iquitos Virus in Traveler Returning to the United States from Ecuador
Source: Emerg Infect Dis. 2024 Nov;30(11):2447–51. doi: 10.3201/eid3011.240708 (PMC11521160; doi:10.3201/eid3011.240708)
Supplement: Appendix — Additional information about Iquitos virus in traveler returning to the United States from Ecuador. [file 24-0708-Techapp-s1.pdf]

Article DOI: https://doi.org/10.3201/eid3011.240708

EID cannot ensure accessibility for supplementary materials supplied by authors. Readers who have difficulty accessing supplementary content should contact the authors for assistance.

Iquitos Virus in Traveler Returning to the United States from Ecuador

Appendix

Sequencing Methods

Two RNA metagenomic sequencing libraries were produced, one with and one without hybridization-based human ribosomal RNA depletion, using previously described methods (*1*). Briefly, samples underwent cDNA synthesis with random primers, Nextera XT library construction (Illumina), and sequencing on an Illumina MiSeq with 150bp paired-end reads, generating a total of 20,679,410 reads.

Reads from both libraries were merged and underwent reference-based assembly using viralrecon v2.6.0, initially with Oropouche virus reference sequences MF926354 (L segment), MF926353 (M), and MF926352 (S). Due to poor assembly of the M segment, reads also underwent de novo assembly using viralrecon v2.6.0. The resulting contigs were evaluated using blastn, the best reference for the M segment was Iquitos virus KF697143, and reference-based assembly was repeated.

Phylogenetic analysis was performed using all available Oropouche virus S and L segment sequences, and all M segment sequences from South American bunyaviruses. Sequences were aligned using MAFFT v7.526, and noncoding regions were trimmed in Geneious Prime v2024.0.5. Maximum-likelihood phylogenetic trees were constructred using IQ-TREE v1.6.12 (*2*), with best-fit models determined using ModelFinder (*3*), and visualized with iTOL (*4*).

References

1. Normandin E, Solomon IH, Zamirpour S, Lemieux J, Freije CA, Mukerji SS, et al. Powassan virus neuropathology and genomic diversity in patients with fatal encephalitis. Open Forum Infect Dis. 2020;7:ofaa392. PubMed https://doi.org/10.1093/ofid/ofaa392

2. Nguyen L-T, Schmidt HA, von Haeseler A, Minh BQ. IQ-TREE: a fast and effective stochastic algorithm for estimating maximum-likelihood phylogenies. Mol Biol Evol. 2015;32:268–74. PubMed https://doi.org/10.1093/molbev/msu300

3. Kalyaanamoorthy S, Minh BQ, Wong TKF, von Haeseler A, Jermiin LS. ModelFinder: fast model selection for accurate phylogenetic estimates. Nat Methods. 2017;14:587–9. PubMed https://doi.org/10.1038/nmeth.4285

4. Letunic I, Bork P. Interactive Tree Of Life (iTOL) v5: an online tool for phylogenetic tree display and annotation. Nucleic Acids Res. 2021;49(W1):W293–6. PubMed https://doi.org/10.1093/nar/gkab301

5. Rojas A, Stittleburg V, Cardozo F, Bopp N, Cantero C, López S, et al. Real-time RT-PCR for the detection and quantitation of Oropouche virus. Diagn Microbiol Infect Dis. 2020;96:114894. PubMed https://doi.org/10.1016/j.diagmicrobio.2019.114894

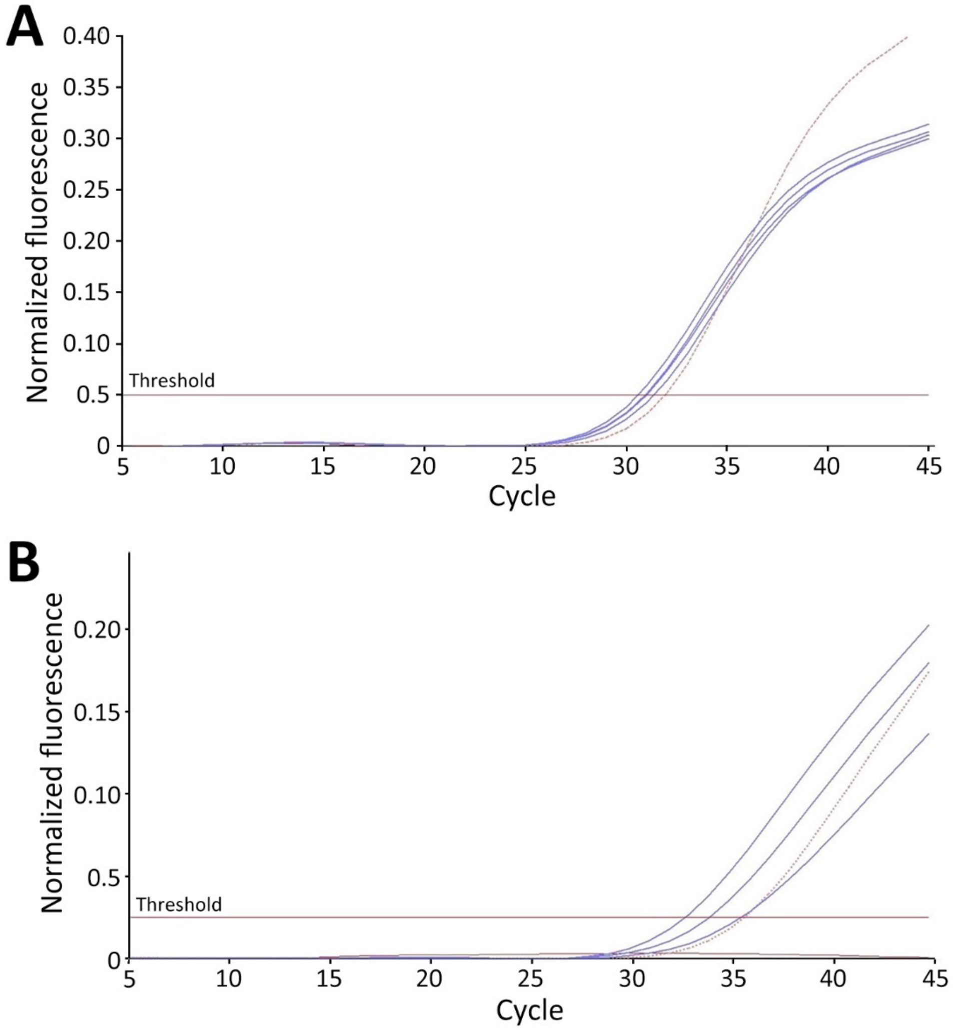

Appendix Figure. Amplification curves for the case-patient sample in two different real-time RT-PCRs. A) Results of testing with a laboratory-developed real-time reverse transcription PCR assay targeting the small genome segment of Oropouche virus and Iquitos virus, from whole blood extracted with the RNA extraction and storage (RNAES) protocol (blue curves) and a positive control (dashed pink). B) Confirmatory real-time reverse transcription PCR results using a published comparator (*5*).
